# Granular Cell Tumor of Rectum: A Very Rare Entity

**DOI:** 10.1155/2017/3795482

**Published:** 2017-01-31

**Authors:** Tagore Sunkara, Savitha V. Nagaraj, Vinaya Gaduputi

**Affiliations:** ^1^Department of Internal Medicine, The Brooklyn Hospital Center, Brooklyn, NY, USA; ^2^Department of Internal Medicine, SBH Health System, Bronx, NY, USA

## Abstract

Granular cell tumors are predominantly benign, occurring more commonly in women, with about 10% developing in the gastrointestinal tract. Rectal location of this tumor is very rare. We herein report one such case of a 61-year-old man with granular cell tumor in the rectum who underwent endoscopic curative resection.

## 1. Introduction

Granular cell tumor (GCT), also known as granular cell myoblastoma or Abrikossoff tumor, was initially reported by Abrikossoff in 1926 [1]. It is a soft tissue neoplasm, arising from Schwann cells [2]. Predominantly a benign tumor, 1-2% of the cases are reported to be malignant [3–5]. It is more commonly seen in women and in the age group of 10–50 years [6–8]. Granular cell tumor can arise in any body site and is most commonly seen in skin, subcutaneous tissue, oral cavity, and gastrointestinal tract [8, 9]. About 10% of the tumors develop in the gastrointestinal tract with esophagus being the most common site and rectum being the rarest [10]. Although there are cases of granular cell tumor in various parts of the gastrointestinal system, there are very few reported cases of granular cell tumor in the rectum, especially in a male patient. We report a rare case of rectal granular cell tumor in a 61-year-old male patient.

## 2. Case Report

**Figure 1:**
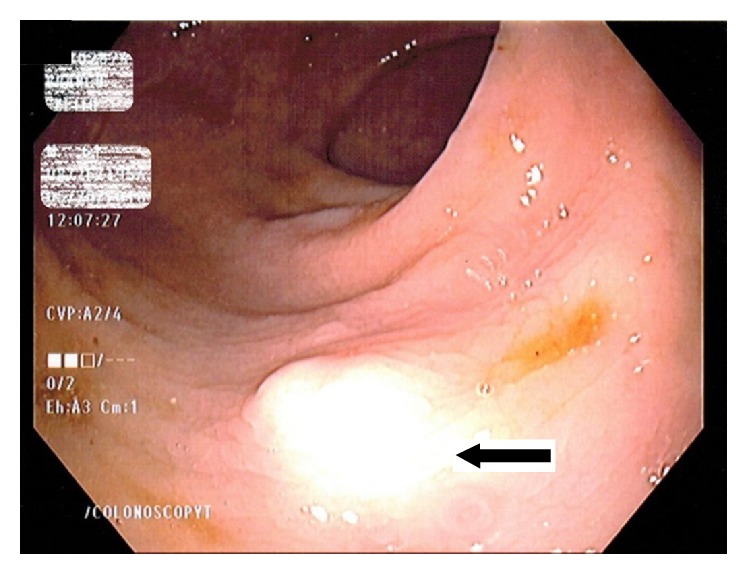
The 4 mm firm nodule visualized in rectum.

**Figure 2:**
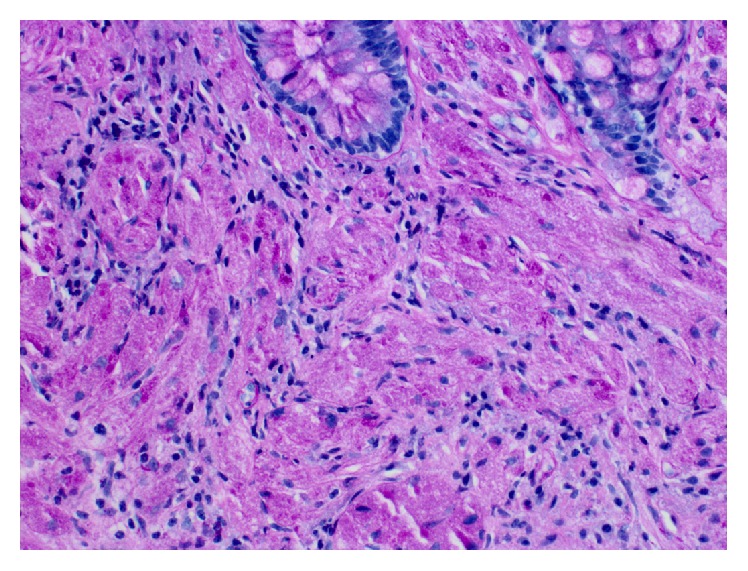
Rectal nodule biopsy (400x) revealing tumor cells arranged in sheets with small round-to-oval nuclei consistent with granular cell tumor on periodic acid-Schiff stain.

**Figure 3:**
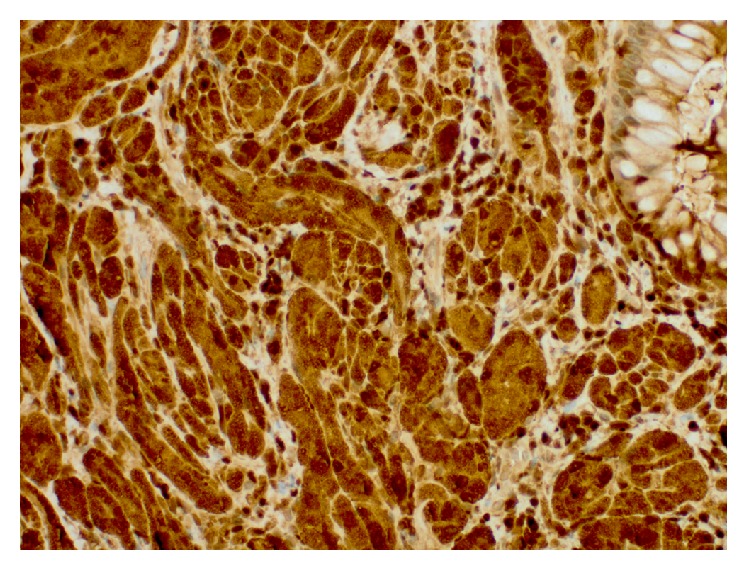
Biopsy revealing positive immunohistochemical staining for S-100 protein.

A 61-year-old man with medical comorbidities of coronary artery disease, congestive heart failure, hypertension, and dyslipidemia presented to the gastroenterology clinic for screening colonoscopy. Patient denied any gastrointestinal related complaints. Screening colonoscopy revealed good bowel preparation with a score of 8 on Boston Bowel Preparation Scale, a 1 cm serrated adenomatous polyp in the transverse colon that was removed with hot snare polypectomy, and a firm 4 mm nodule in the rectum that was removed with biopsy forceps (Figure 1). Biopsy of the rectal nodule revealed a granular cell tumor with positive periodic acid-Schiff (PAS) staining (Figure 2). Immunohistochemical staining for S-100 protein was positive as well (Figure 3). A subsequent rectal endoscopic ultrasound (EUS) confirmed complete removal of the tumor.

## 3. Discussion

Granular cell tumor (GCT) is a neoplasm of mesenchymal origin. It is thought to originate from the Schwann cells due to its positive staining for S-100, myelin, and myelin associated glycoprotein [2]. Histologically, GCT is comprised of large polygonal cells with eosinophilic cytoplasm containing PAS positive granules, abundant lysosomes, and small and uniform nuclei [11, 12]. It is more common in females compared to males and occurs predominantly in the age group of 10–50 years. It can occur in any part of the body but in the gastrointestinal tract, esophagus is the commonest location.

GCT commonly presents as a solitary mass, although some may present with multiple tumors in multiple locations [11]. In the gastrointestinal tract, tumor can present as a painless, nonulcerated nodule or a yellowish-gray sessile polyp with firm consistency. It is often found incidentally and needs to be differentiated from other submucosal tumors such as stromal tumor, carcinoid, steatoma, or smooth muscle tumor. On endoscopic ultrasound (EUS), GCT appears as small (95% < 2 cm), hypoechoic, solid, homogenous tumor with invasion of the inner and/or outer layers of the gastrointestinal tract (mucosa/submucosa) [13]. GCT is commonly misdiagnosed as carcinoid tumor [14], with both tumors being mucosal or submucosal in location and having similar endoscopic findings. The carcinoid tumor arises from the enterochromaffin cells of the gastrointestinal tract and can be differentiated histologically and chemically from GCT [15].

GCT is mostly a benign tumor; however 2% of them can be malignant. A tumor greater than 3 cm or rapid tumor growth and ulceration raise a suspicion for malignant transformation [3, 4]. Fanburg Smith and colleagues proposed six criteria based on tumor histopathology to determine tumor malignancy and prognostic factors: cell necrosis, spindling, pleomorphism, increased mitotic activity (>2 mitoses/10 HPF at 200x magnification), vesicular nuclei with large nucleoli, and high nuclear to cytoplasmic ratio. Neoplasms were classified as malignant if they met three or more of these criteria, atypical if they met one to two of these criteria, and benign if they displayed only focal pleomorphism and did not fulfill any other criteria [16].

Definitive diagnosis of GCT can be made by endoscopic biopsy and histopathological studies. The mainstay of treatment for a benign GCT, as was with our patient, is endoscopic resection. Different methods of endoscopic resections (mucosal and submucosal resections) are widely used and some resections with elastic band ligation have been reported [17]. For asymptomatic and smaller tumors, endoscopic surveillance may be sufficient [12]. Endoscopic ultrasound can be further performed to evaluate tumor invasion and assess complete tumor excision. Surgical resection with adequate margins can be reserved for large, malignant, and multifocal tumors invading the outer layers.

## 4. Conclusion

Granular cell tumors of gastrointestinal tract are rare entities with very few reports of rectal location. Although it is mostly a benign tumor, an astute clinician must be aware of possible malignant variants and the features of such lesions. It is equally important to differentiate granular cell tumor from other endoscopically similar mucosal and submucosal tumors of the rectum. Most gastrointestinal granular cell tumors are amenable for endoscopic resection which is often curative.

## Data Access

Data relating to this case report is available from the corresponding author upon request.
